# Complete Freund’s adjuvant as a confounding factor in multiple sclerosis research

**DOI:** 10.3389/fimmu.2024.1353865

**Published:** 2024-02-15

**Authors:** Milica Lazarević, Suzana Stanisavljević, Neda Nikolovski, Mirjana Dimitrijević, Đorđe Miljković

**Affiliations:** Department of Immunology, Institute for Biological Research “Siniša Stanković” - National Institute of Republic of Serbia, University of Belgrade, Belgrade, Serbia

**Keywords:** multiple sclerosis, experimental autoimmune encephalomyelitis, complete Freund’s adjuvant, antigen, pain

## Abstract

Complete Freund’s adjuvant (CFA) is used as a standard adjuvant for the induction of experimental autoimmune encephalomyelitis (EAE), the most commonly used animal model in multiple sclerosis studies. Still, CFA induces glial activation and neuroinflammation on its own and provokes pain. In addition, as CFA contains Mycobacteria, an immune response against bacterial antigens is induced in parallel to the response against central nervous system antigens. Thus, CFA can be considered as a confounding factor in multiple sclerosis–related studies performed on EAE. Here, we discuss the effects of CFA in EAE in detail and present EAE variants induced in experimental animals without the use of CFA. We put forward CFA-free EAE variants as valuable tools for studying multiple sclerosis pathogenesis and therapeutic approaches.

## 1 Introduction

Experimental autoimmune encephalomyelitis (EAE) is an animal model of the central nervous system (CNS) autoimmune diseases. It is mostly used to study the pathogenesis of multiple sclerosis and to explore therapeutic approaches for the disease (1). EAE was invented by Rivers and colleagues in 1933 as a consequence of studying the neurological side effects of vaccination against rabies (2). For more details on the timeline of the origin and development of EAE, readers are referred to a comprehensive review by Alan G. Baxter (3). Complete Freund’s adjuvant (CFA) was introduced as an essential component for EAE induction as early as 1947 (4, 5), just 5 years after its invention (6). CFA is used in most variants of EAE, including the most prominent ones, such as myelin oligodendrocyte glycoprotein (MOG)_35–55_–induced chronic EAE in C57BL/6 mice, proteolipid protein (PLP)_139–151_–induced relapsing-remitting EAE in Swiss Jim Lambert (SJL) mice, and myelin basic protein (MBP)–induced monophasic acute EAE in Lewis rats. Moreover, T-cell lines produced for adoptive transfer in passive EAE are originating from animals immunized with CNS antigens emulsified in CFA (7).

**Figure 1:**
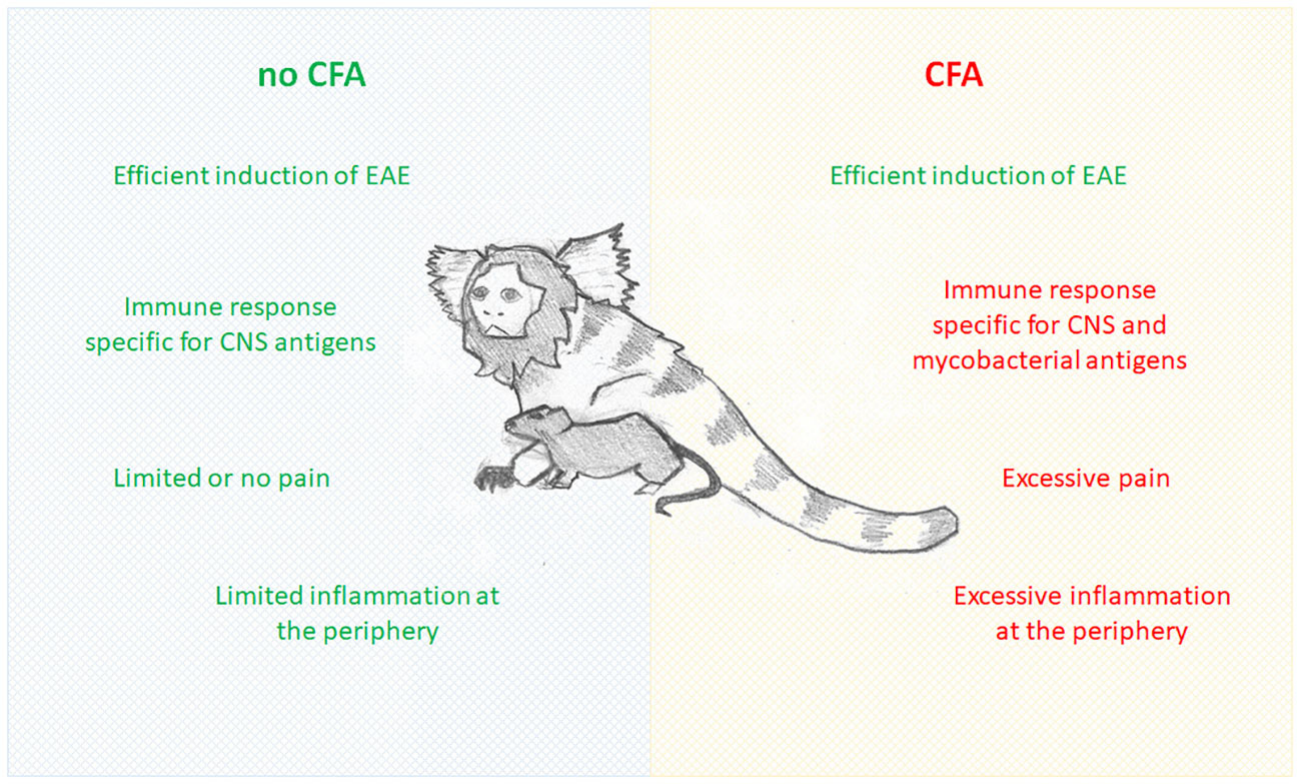
Comparison of EAE induced without and with CFA.

CFA is a suspension of desiccated heat-inactivated mycobacterium (most commonly used is *M. tuberculosis*, followed by *M. butyricum*) in paraffin oil and mannide monooleate. It has been regularly used as an adjuvant of choice for induction of autoimmune disorders in experimental animals (8). CFA extends injected autoantigen lifetime, galvanizes the effective delivery of the antigens to the immune system, and stimulates the innate compartment of the immune system (8), which, in turn, results in the effective induction of autoimmune disorders in experimental animals, including EAE. Still, in studies where EAE is used as a multiple sclerosis model, CFA introduces numerous confounding effects, including immune reactivity against antigens unrelated to the CNS, activation of glia independent of the autoimmune process, and induction of pain (**Figure 1**). These and other confounding effects of CFA are discussed in detail in the following sections.

## 2 Confounding effects of CFA in EAE

### 2.1 Mycobacteria

One of the confounding effects of CFA arising from the presence of mycobacteria is the stimulation of lymphocytes specific for non-CNS antigens that contribute to the inflammatory response in EAE (8). As an example, it has been reported that lymphocytes obtained from the lymph nodes draining the site of immunization or from the CNS of EAE rats immunized with MBP + CFA exhibited reactivity against mycobacterial purified protein derivative (PPD) (9). Moreover, the addition of a PPD-specific T-cell line to MBP-specific T cells in passive EAE induction resulted in increased blood-brain barrier disruption, suggesting that PPD-specific T cells contribute to the CNS autoimmunity in EAE (10). On the contrary, reactivity to mycobacterial antigens homologous to mammalian proteins, such as heat-shock proteins (HSPs), may interfere with EAE induction. Indeed, prophylactic application of mycobacterial HSP was shown to inhibit the development of EAE (11, 12).

Mycobacteria are intracellular bacteria, and the dominant immune response against them is performed by activated T helper 1 (Th1) cells. Therefore, there is a concern that CFA skews immune response toward Th1 and away from Th17 arm. Both Th populations are involved in multiple sclerosis pathogenesis, and each population has been associated with different clinical expressions of the disease and/or different mechanisms of effector actions within the CNS (13, 14). Still, CFA does not contain live mycobacteria, and it is more likely that it shapes immune response through mycobacterial pathogen-associated molecular patterns, such as muramyl dipeptide (MDP), trehalose dimycolate (TDM), and lipoarabinomannan (LAM), which are recognized by nucleotide oligomerization domain (NOD), C-type lectin-type receptors, or Toll-like receptor 2 (TLR2), respectively (8, 15–17). Whereas TLR2 stimulation enhances production of cytokines interleukin (IL)-12, interferon (IFN)--γ, and IL-18, promoting Th1 differentiation, TDM induces production of TNF, IL-6, and CXCL2, promoting development of both Th1 and Th17 cells (8, 17). Similar to TDM-induced stimulation, acute NOD2 activation by MDP results in induction of TNF, IL-12, IL-6, IL-8, and IL-10 (18). Th1-dominated cytokine pattern is also induced through TLR9 stimulation by unmethylated 5′-C-phosphate-G-3′ (CpG) oligodeoxynucleotides and TLR4 stimulation by mycobacterial HSP (19–21). Thus, it seems that CFA is able to produce both Th1 and Th17 responses. As an example, both Th1 and Th17 T cells are present in the CNS in EAE induced in Dark Agouti (DA) rats with spinal cord homogenate (SCH) + CFA (22). Still, predominance of Th1 response cannot be excluded as a confounding factor of CFA.

The other important question is if mycobacteria are involved in the aetiology and pathogenesis of multiple sclerosis. There were studies on the role of mycobacterial HSP70 in multiple sclerosis (23, 24), not only on the human zoonotic infection with *M. avium* subsp. *paratuberculosis* as a cause of multiple sclerosis (25, 26) but also on the protective role of vaccination with attenuated strain of *M. bovis* (BCG vaccine) on disease activity in patients with multiple sclerosis (27, 28). In short, it seems that *M. avium* subsp. *paratuberculosis* might be a causal agent in genetically predisposed individuals that appear to be restricted to certain human populations, such as those of Sardinia and Japan. As for BCG, epidemiological data support its protective role in multiple sclerosis, but the mechanisms behind its effects are still elusive. For more details on the possible role of mycobacteria in multiple sclerosis etiopathogenesis, readers are referred to a review by Cossu and colleagues (29). Thus, it seems that there is insufficient evidence on the role of mycobacteria in multiple sclerosis pathogenesis to support the use of CFA in EAE.

In addition, one should be careful when studying gut/lung microbiota role in the pathogenesis of the CNS autoimmunity using EAE induced with CFA, as there are studies showing that CFA is affecting microbiota in experimental animals (30, 31). Overall, as different findings demonstrated diverse relationship between mycobacteria and multiple sclerosis, the presence of mycobacterial components in CFA during EAE induction could potentially serve as a disease-modifying factor. This could pose challenges in conclusively identifying the precise pathological mechanisms driving the CNS autoimmunity and in evaluating potential mechanisms for multiple sclerosis therapy.

### 2.2 Pain, glial activation, and neuroinflammation

CFA is known to induce pain in experimental animals (8). Continual release of antigens from the oily deposit induces a delayed hypersensitivity reaction characterized by intense inflammation and hyperalgesia at the injection site (8). Noted responses to CFA include local acute and chronic inflammation, granulomatous reactions, skin ulceration, local abscess, and sloughing. Systemic reactions include diffuse systemic granulomas resulting from the migration of the oil emulsion, adjuvant-related arthritis, and chronic wasting disease (32). Inflammatory pain is influenced by various modulators, including neurotransmitters, receptors, ion channels, and signaling pathways (33). In a study exploring effects of CFA on various metabolites, a decrease of arginine levels and an increase in histidine, phenylalanine, and tyrosine levels were found in response to CFA injection (34). These changes in amino acid levels have been associated with alterations in neurotransmitter levels and, subsequently, with potentiation of chronic inflammatory pain (34). Additional mechanisms involved in CFA-induced pain include the production and release of prostaglandin E_2_, NO, leukotriene B_2_, TNF, IL-2, and IL-17 (35). These mediators contribute to synovitis, polyarticular inflammation, bone resorption, periosteal bone proliferation, and consequently to joint degeneration (36).

CFA is typically used to induce peripheral inflammation that can subsequently affect the CNS. Peripheral inflammation induced by CFA leads to the release of inflammatory mediators, including cytokines that can affect the blood-brain barrier and influence communication between the immune system and the CNS. Indeed, it was previously demonstrated that the permeability of the blood-brain barrier increased after CFA administration (37). This can affect both glial cells and neurons and ultimately induce pain. Accordingly, CFA-induced pain in experimental animals is paralleled with the activation of glia and the production of inflammatory mediators in the spinal cord (38). Microglia and astrocytes are recognized as active participants in the initiation and maintenance of pain facilitation triggered by inflammation and damage to peripheral tissues, peripheral nerves, spinal nerves, and spinal cord (39–42). Upon activation, glial cells release a variety of mediators, including proinflammatory cytokines that can enhance pain transmission by activating and sensitizing neurons (41–44). In turn, activated neurons can have reciprocal effects on glial cells, thus maintaining persistent inflammation and prolonged pain sensitization (45). Specifically, increased expression of IL-1β and IL-1RI was found in glial cells and sensory neurons in an articular arthritis model induced with CFA (46). Furthermore, intraplantar administration of CFA led to elevated expression of microglial markers (Mac-1, CD11b/c, TLR4, and CD14) in the spinal cord and brain during all stages of inflammation (47). In contrast to microglia, increased expression of astrocytic markers, glial fibrillary acidic protein (GFAP) and S100 calcium-binding protein B (S100B), was detected only at the later stages, indicating delayed astrocytic activation. Having in mind all before mentioned, immunization with CFA represents a standard model for studying pain (48).

Considering the wellbeing of experimental animals, the ability of CFA to cause inflammatory pain in experimental animals is problematic *per se* and should be avoided to prevent unnecessary suffering and additional distress in animals. Furthermore, the enduring inflammatory pain induced by CFA interferes with various classical tests assessing exploratory behavior, stress coping, and naturalistic behavior. A systematic review and meta-analysis, encompassing numerous experiments with hundreds of mice and rats, distinctly revealed that CFA markedly reduces exploratory behavior and heightens immobility in the tail suspension test (49). The most pronounced negative impact was observed in naturalistic behaviors like burrowing and wheel running. Finally, pain induced by CFA interferes with the studies of pain caused by CNS autoimmunity and, consequently, with translation to multiple sclerosis. Neuropathic pain is a common symptom in patients with multiple sclerosis, affecting between 28 and 87% of individuals (50, 51). It stems from damage of the central or peripheral somatosensory systems, including the hyperexcitability of neurons within pain pathways (52). As CFA induces pain on its own, EAE induced with CFA is not a reliable animal model for analyzing the mechanism underlying chronic neuropathic pain frequently registered in patients with multiple sclerosis.

## 3 EAE models without CFA

EAE can be induced in monkeys and rats with IFA (incoplete Freund’s adjuvant) as adjuvant or even without adjuvant at all. Although EAE cannot be induced in mice without adjuvant, transgenic mice develop EAE without induction (*vide infra*). The list of actively induced EAE variants in experimental animals without adjuvant or with IFA is provided in **Table 1**. Importantly, the idea of using CFA-free animal models in the study of CNS autoimmunity is not novel, having been proposed by pioneers in the field, Levine and Wenk, some 60 years ago (65). These authors were able to show that EAE can be induced by intracutaneous injection of neural tissue homogenates without adjuvant in various strains of rats, with Lewis rats being the most susceptible. In addition, they tested SCH of different origins, including various rodents, dogs, bovines, guinea pigs, and even humans. Their results showed that guinea pig and rat SCH were by far the most efficient.

**Table 1:** Active EAE models with IFA or no adjuvant.

	Model	Antigen	Adjuvant	Clinical Course	Reference
Monkeys	Marmosets	rhMOG_20-50_ rhMOG_14-36_ rhMOG_34-56_ rhMOG_74-96_	IFA	Chronic/progressive	(53–55)
	Marmosets Rhesus Cynomolgus	rhMOG_1-125_	IFA	Monophasic/relapsing/ progressive	(56, 57)
Rats	DA	rSCH	IFA	Protracted/relapsing	(58)
	DA Lewis	rSCH	IFA	Relapsing	(59)
	Lewis	rrMOG_1-125_	IFA	Chronic/progressive	(60)
	DA	rmMOG_1-116_	IFA	Chronic/relapsing	(61, 62)
	DA SD	rhMOG_1-125_	IFA	Benign to progressive	(63, 64)
	Lewis CD F	gpSCH	No IFA	NA	(65)
	DA	rSCH gpSCH	No	Chronic/relapsing	(66–68)

CD F, Fischer; DA, Dark Agouti; EAE, experimental autoimmune encephalomyelitis; gp, guinea pig; IFA, incoplete Freund’s adjuvant; MOG, myelin oligodendrocyte glycoprotein; NA – not addressed; r, rat; rh, recombinant human; rm, recombinant mouse; rr, recombinant rat; SCH, spinal cord homogenate; SD, Sprague Dawley.

DA rats were not investigated in their study, but we were able to convincingly demonstrate that these rats are also highly susceptible to CFA-free EAE (66–68). Classically, EAE in DA rats is induced with SCH (either guinea pig or rat) emulsified in CFA (69–71). Moreover, if immunization of DA rats is performed with SCH mixed with carbonyl iron, then strong and highly lethal EAE is induced (72). Still, EAE is easily induced in DA rats by rat SCH immunization even without adjuvant. The incidence higher than 90% and the clinical course that is prolonged in comparison to SCH + CFA immunization was determined in DA rats immunized with SCH (67). Infiltrates were observed in both the white and the gray matter of the spinal cord and also in the brain (68). This model has proven useful for evaluating the therapeutic efficacy of novel agents (73, 74). We are currently investigating cellular and molecular mechanisms behind the pathogenesis of CNS autoimmunity in this model in detail. We are particularly interested in the effects of immune cells on various brain structures, such as the cortex, the cerebellum, and the hippocampus. Moreover, antigen specificity of T and B cells activated by immunization with SCH in DA rats is underway.

Furthermore, DA rats immunized with rat SCH and IFA develop severe protracted and relapsing EAE characterized by extensive demyelinating inflammatory lesions and autoreactivity to the rat-specific neuro antigens MOG, MBP_69–87_, and MBP_87–101_ (58). Axonal loss in DA rats immunized with MOG in IFA correlates with clinical severity and number of relapses (61). A similar mechanism of inflammatory demyelination involving different components of endoplasmic reticulum stress has been proposed for demyelination in both the MOG + IFA rat EAE model and in multiple sclerosis (62). Recently, a novel low-dose MOG + IFA–induced EAE rat model was introduced in DA and SD rats to investigate pain with minimal motor impairment/disability and to find a potential treatment for multiple sclerosis–related pain (63, 64). The high susceptibility of DA rats to EAE was also confirmed by the induction of severe disease after injection of an encephalitogen in Titermax, an adjuvant consisting of the block copolymer CRL-8300, squalene, and a sorbitan monooleate (75).

EAE in marmosets immunized with rhMOG peptides in IFA is characterized by demyelinating cortical gray matter and prevalent white matter lesions (53–55). Interestingly, the cross-reactivity of MOG-specific T cells with the effector memory cells that control latent CMV infection was determined in rhesus monkeys (76). Although, this cross-reactivity was not investigated in marmosets, the possibility exists that CMV infection, which is common in these monkeys, contributes to the rhMOG peptide + IFA–induced EAE model. The variable clinical presentation of EAE in monkeys is related to inflammation and demyelination in the CNS (56), whereas the occurrence of an early anti-rhMOG IgM response is associated with a more severe disease course (76). However, the purity of the antigen is critical for the development of a mild form of EAE using IFA, as low traces of LPS in rhMOG can increase the severity of clinical and histologic features of the disease in cynomolgus monkeys (57).

Finally, resistance of mice to EAE induction with MOG_35–55_ or PLP_139–151_ in IFA can be abolished by addition of the bacterial component peptidoglycan from *S. aureus* (77) or by co-injection of pertussis toxin (78, 79), respectively. EAE can also be induced in mice with MOG_35–55_ and quillaja bark saponin as an adjuvant followed by pertussis toxin injections (80, 81). Microbial products seem to be essential for efficient antigen-presenting cell activity and, consequently, for the induction of CNS autoimmunity in mice (77–79). Thus, it can be concluded that active EAE induction in mice is not possible without adjuvant or with IFA as the only adjuvant. However, there are transgenic mouse models that develop EAE without any immunization, such as double-transgenic TCR^MOG35–55^ × IgH^MOG^ C57BL/6 or non-obese diabetic (NOD) mice (82–84) and TCR^MOG92–106^ transgenic SJL mice (85, 86). These models are very useful for studying the pathogenesis of CNS autoimmunity yet with limitations introduced by intrinsic restriction in the specificity of their antigen recognition receptors.

Importantly, different models presented in **Table 1** cover the full spectrum of multiple sclerosis expression, from benign/mild, through the most common relapsing-remitting, and to the chronic and progressive forms. For a comprehensive understanding of the complexity of clinical manifestations of multiple sclerosis and their significance for the pathogenesis and treatment of the disease, please refer to a review by Confavreux and Vukusic (87). This versatility of the CFA-free animal models makes them useful for studying and addressing specific questions related to different clinical forms of multiple sclerosis.

## 4 Discussion

Numerous variants of EAE are available for the investigation of multiple sclerosis. Given the diversity in the pathogenesis of multiple sclerosis as well as the elusive etiology of the disease, each of the EAE variants is a valuable tool for the studies. We strongly support the use of CFA-free EAE variants as complementary models in multiple sclerosis studies. This approach can help to eliminate the potential effects of CFA, which were described in detail above. Interestingly, there were important early reports showing that EAE can be induced in rats by immunization with an emulsion made of CFA and lung homogenate (88). In addition, a demyelinating disease was induced in Syrian hamsters and guinea pigs by liver homogenate emulsified in CFA (89). It seems unlikely that CNS and lung or liver share some common antigens that could serve as autoantigens in CNS autoimmunity. It is more likely that CFA leads to neuroinflammation independent of the presence of CNS antigens in the reported studies. This is one more reason to consider CFA as a confounding factor in multiple sclerosis studies based on EAE. Thus, we propose that CFA-free animal models should be used more frequently in the exploration of multiple sclerosis pathogenesis and therapeutic opportunities. Contemporary studies of multiple sclerosis should provide a means to prevent demyelination and neurodegeneration and to promote remyelination and neuroregeneration. We strongly suggest CFA-free EAE variants for such studies as they are superior to CFA-based EAE variants, which are discredited by the weaknesses introduced into the model by the use of CFA, as discussed above.

## Author contributions

ML: Writing – original draft, Writing – review & editing. SS: Writing – original draft, Writing – review & editing. NN: Writing – original draft, Writing – review & editing. MD: Writing – original draft, Writing – review & editing. ĐM: Conceptualization, Writing – original draft, Writing – review & editing.
